# Deciphering the antiviral mechanisms of Fangqin Qinggan decoction against influenza A virus: a multi-omics and machine learning approach

**DOI:** 10.1186/s13020-025-01211-0

**Published:** 2025-10-05

**Authors:** Huan Lei, Hao Zhang, Yixi Xu, Lianjiang Hu, Bin Zhang, Hao Zhou, Ping Wang, Simin Chen, Shijun Xu

**Affiliations:** 1 State Key Laboratory of Southwestern Chinese Medicine Resources, Chengdu University of Traditional Chinese Medicine, Chengdu, 611137 People’s Republic of China; 2 School of Pharmacy, Chengdu University of Traditional Chinese Medicine, Chengdu, 611137 People’s Republic of China; 3 Institute of Material Medica Integration and Transformation for Brain Disorders, Chengdu University Traditional Chinese Medicine, Chengdu, 611137 People’s Republic of China; 4 School of Pharmacy, Chengdu University, Chengdu, 611137 People’s Republic of China; 5 College of Medical Technology, Chengdu University of Traditional Chinese Medicine, Chengdu, 611137 People’s Republic of China

**Keywords:** Influenza A virus, Fangqin Qinggan decoction, Machine learning, Multi-omics, Viral replication

## Abstract

**Background:**

Influenza A virus (IAV) infection poses a significant global health burden, contributing to high morbidity and mortality in both humans and animals through rapid viral transmission and dysregulated inflammatory responses. Fangqin Qinggan Decoction (FQ-01), a traditional Chinese medicine (TCM) formula, has demonstrated clinical efficacy in treating viral upper respiratory infections, however, its underlying therapeutic mechanisms remain poorly understood.

**Methods:**

The therapeutic efficacy and mechanisms of FQ-01 against IAV infection were comprehensively investigated using a multidisciplinary approach, including in vivo murine models, histopathological (H&E staining), RT-qPCR, immunohistochemistry (IHC), network pharmacology, weighted gene co-expression network analysis (WGCNA), machine learning (LASSO), transcriptomics, metabolomics, molecular docking and molecular dynamics (MD) simulation.

**Results:**

FQ-01 significantly improved survival rates, reduced clinical mortality, and mitigated pulmonary inflammation in an IAV-infected mice while suppressing viral replication. Integrated bioinformatics and LASSO regression analyses identified 20 genes associated with FQ-01’s antiviral effects, with *Myd88* and *Ccl5* emerging as key targets. Transcriptomic profiling of murine lung tissues further validated these genes as critical mediators of FQ-01’s therapeutic action. Spearman correlation analysis revealed strong associations between *Myd88*/*Ccl5* expression and serum/lung metabolites, particularly 3-indolyl sulfate and inosine. Subsequent in vivo RT-qPCR and IHC validation, molecular docking, and MD simulations confirmed that FQ-01 exerts its anti-IAV effects by inhibiting *Myd88* and *Ccl5* expression.

**Conclusions:**

Our findings elucidate the molecular mechanisms underlying FQ-01’s therapeutic potential against IAV infection, highlighting *Myd88* and *Ccl5* as promising targets for antiviral and anti-inflammatory interventions. This study provides a foundation for further exploration of TCM-based strategies in combating influenza and related respiratory infections.

**Graphical Abstract:**

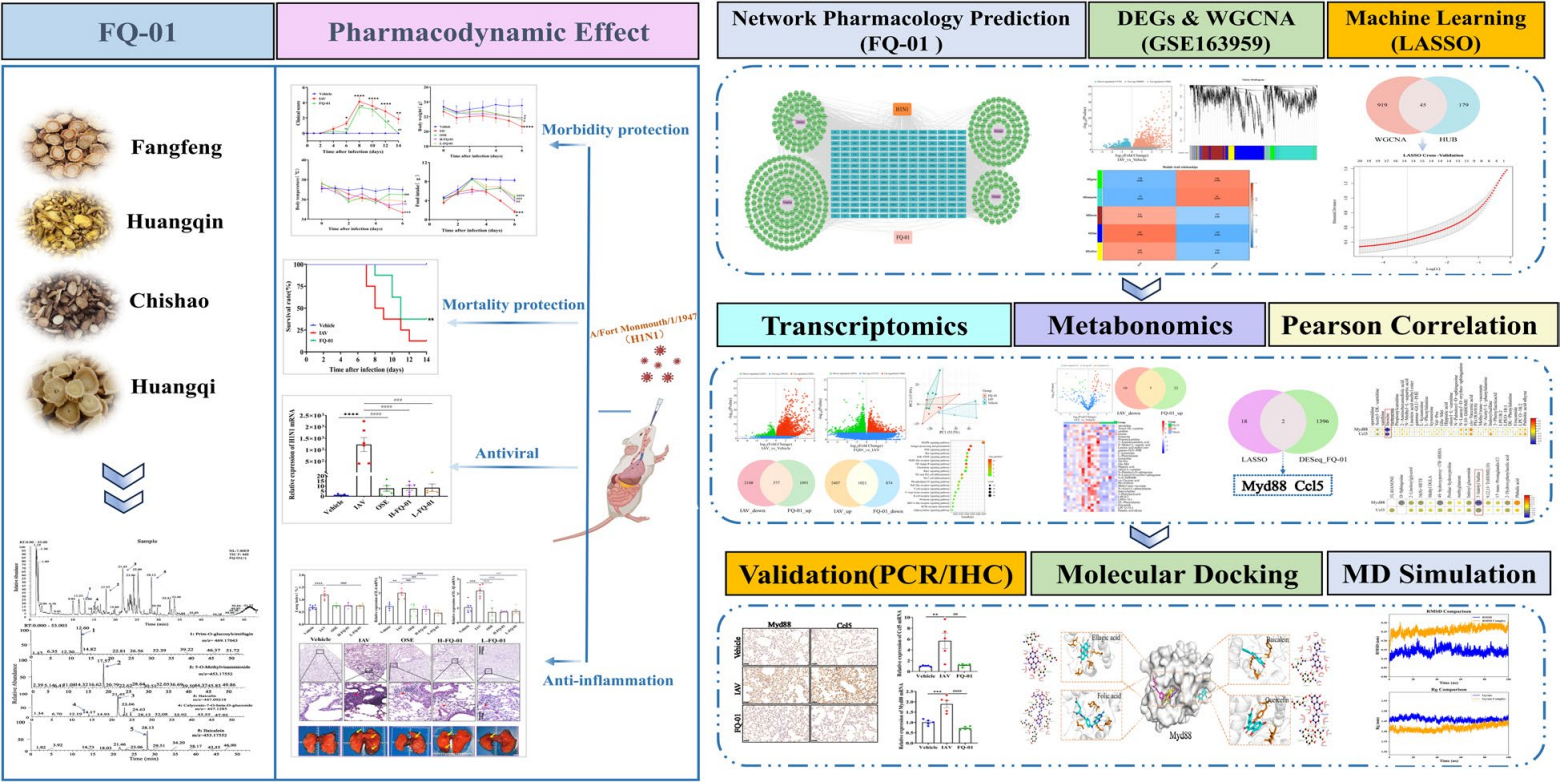

**Supplementary Information:**

The online version contains supplementary material available at 10.1186/s13020-025-01211-0.

## Introduction

Influenza A virus (IAV) poses a persistent global health challenge, leading to substantial morbidity and mortality from respiratory infections, with an estimated 300,000 annual deaths attributable to influenza-related complications [1]. The virus’s high mutation rate, driven by antigenic drift and reassortment (antigenic shift), facilitates immune evasion and complicates the development of broadly effective antiviral strategies. Although current therapies, including neuraminidase inhibitors (e.g., oseltamivir) and RNA polymerase inhibitors (e.g., baloxavir marboxil), remain frontline treatments, their efficacy is increasingly compromised by the emergence of drug-resistant viral strains, the narrow therapeutic window and their limited prophylactic utility [2, 3]. These challenges underscore an urgent need for novel therapeutic interventions to combat IAV infections.

Traditional Chinese Medicine (TCM) has been extensively employed for centuries in managing respiratory infections, representing a rich repository of potential antiviral agents. Fangqin Qinggan Decoction (FQ-01), a classical TCM formula, originates from Huangqi Chifeng Decoction as documented in the Qing Dynasty medical treatise *Yilin Gaicuo* (Corrections of Errors in Medical Classics) by Wang Qingren [4]. This formula has demonstrated its efficacy in treating viral upper respiratory infections [5]. FQ-01 comprises four principal medicinal herbs: Fang Feng (*Saposhnikovia divaricata* (Turcz. ex Ledeb.) Schischk), Huang Qin (*Scutellaria baicalensis* Georgi), Chi Shao (*Paeonia lactiflora* Pall), and Huang Qi (*Astragalus mongholicus* Bunge). Contemporary pharmacological studies have identified multiple bioactive compounds in these herbs that exhibit potent antiviral activity, anti-inflammatory effects, and immunomodulatory properties [6–9]. Despite its clinical application, the therapeutic efficacy of FQ-01 against influenza virus and its underlining mechanisms remain to be fully elucidated.

Recent advances in bioinformatics have revolutionized biomedical research, with integrated machine learning approaches and multi-omics technologies now playing a pivotal role in biomarker discovery and therapeutic target identification [10, 11]. In the present study, we first demonstrated the potent antiviral and anti-inflammatory properties of FQ-01 through systematic pharmacological evaluation. Building on these findings, we implemented an innovative multi-modal research strategy combining: (1) computational bioinformatics, (2) machine learning algorithms (LASSO regression), (3) transcriptomic profiling, and (4) untargeted metabolomics to comprehensively characterize FQ-01’s bioactive components and elucidate its molecular mechanisms against IAV. Our integrated analysis revealed that FQ-01 exerts therapeutic effects through coordinated modulation of key immune regulators (*Myd88* and *Ccl5*) and metabolic pathways involving 3-indoxyl sulfate and inosine.

Notably, studies have demonstrated that upon influenza virus infection, the recognition of viral components by Toll-like receptors (TLRs) activates downstream signaling, leading to the recruitment of Myd88 [12]. This in turn promotes the activation of the NF-κB transcription factor, which induces robust secretion of pro-inflammatory cytokines (such as IL-1β, IL-6, and TNF-α) [13] and chemokines (including Ccl5, Ccl2, and Cxcl10) [14]. Based on this mechanism, our integrated multi-omics analysis suggests that FQ-01 may alleviate excessive inflammatory response and pathological damage caused by influenza virus infection through modulating key signaling molecules such as Myd88 and Ccl5, thereby regulating innate immune and inflammatory responses.

## Materials and methods

### Preparation of FQ-01 extract

The FQ-01 formulation was prepared according to traditional protocols with standardized modifications. Dried crude herbs [Fang Feng (*Saposhnikovia divaricata* Schischk), Huang Qin (*Scutellaria baicalensis* Georgi), Chi Shao (*Paeonia lactiflora* Pall), and Huang Qi (*Astragalus mongholicus* Bunge)] were combined in a precise ratio of 5:9:5:9 (w/w). The herbal mixture was initially soaked in 15 volumes (v/w) of distilled water for 30 min at room temperature, followed by decoction at 100 °C for 60 min. The resulting extract was filtered through sterile gauze (4-layer, 100-mesh) to obtain the primary filtrate. To maximize extraction efficiency, the residue underwent two subsequent extractions (10 and 5 volumes respectively), each with identical boiled duration (60 min). All filtrates were combined and subjected to centrifugation (3000 rpm, 5 min, 25 °C) to remove particulate matter. The supernatant was further clarified through a buchner funnel (Whatman No. 1 filter paper) under vacuum. The pooled extract was concentrated to a final volume of 500 mL using a rotary evaporator (40 °C, 0.09 MPa) and lyophilized to yield FQ-01 powder. Detailed information on the quality control of FQ-01 is provided in Supplementary data 1 and Supplementary Fig. 1.

### High-Resolution mass spectrometry (HRMS)

HRMS was performed using a Q Exactive mass spectrometer equipped with a Thermo Accucore C18 column (100 mm × 3 mm, 2.6 µm). Electrospray ionization (ESI) was applied in both positive and negative ion modes. Data acquisition and analysis were conducted using Xcalibur software.

### Virus and animals

The influenza A virus (H1N1 strain A/Fort Monmouth/1/1947) was generously provided by Professor Yang Falong’s laboratory at Southwest Minzu University. The virus was propagated in 9- to 10-day-old embryonated chicken eggs, and its virulence was determined using a 0.5% chicken red blood cell hemagglutination assay. The virus stock, with a titer of ≥ 2⁷, was maintained at − 80 °C. Balb/c mice (females: 20 ± 2 g; Males: 22± 2 g) were obtained from Beijing Spikefu Biotechnology Co., Ltd. The approval for all experimental procedures was granted by the Animal Care and Use Committee at the Industrial Antibiotics Institute, Chengdu University (Approval No.: SIIA20230901) and conducted in accordance with ethical guidelines for the humane treatment of animals.

### Infection and FQ-01 intervention

For infection studies, the virus stock was serially diluted (10⁻^1^ to 10⁻⁶) in phosphate-buffered saline (PBS) and administered intranasally to Balb/c mice. Mice were randomly assigned to five groups (n = 6): The groups were as follows: the Vehicle group, which received PBS (30 µL/mouse) intranasally; the IAV group, which was infected with 0.8 LD₅₀ of H1N1 virus; the High-dose FQ-01 group (H-FQ-01), which was treated with 4.2 g/kg/day FQ-01; the Low-dose FQ-01 group (L-FQ-01), which received 2.1 g/kg/day FQ-01; and the Oseltamivir phosphate group (OSE), which was treated with 2.5 mg/kg/day oseltamivir. Treatments were administered at 24 h post-infection for five consecutive days. Mice were monitored for 14 days to assess clinical symptoms, and mortality was recorded. The median lethal dose (LD₅₀) was determined by the Reed & Muench method [15]. For subsequent mechanistic studies, female mice were divided into three groups: Vehicle, IAV, and FQ-01 (4.2 g/kg/day). Mice were euthanized 24 h post-infection for lung and serum sample collection, which were then subjected to further analysis.

### Lung histopathology and immunohistochemical analysis

Following euthanasia, the lungs of the mice were excised, rinsed with saline, and dried. The formula for calculating the lung index is: (lung weight/body weight) × 100%. Clinical symptoms, including ruffled fur, hunched posture, and reduced activity, were scored daily based on established criteria [16]. The left lung lobe was fixed in 4% paraformaldehyde for 72 h, followed by graded ethanol dehydration. The tissue was embedded in paraffin, and sectioned into 5 µm sections. These sections were then processed with hematoxylin and eosin (H&E) staining and scanned. A blinded pathologist evaluated five high-power fields (40 × ) per slide, and the mean pathological score was calculated. Higher scores corresponded to more severe tissue damage [16, 17].

For immunohistochemical analysis, lung sections were deparaffinized using xylene and ethanol, followed by antigen retrieval with EDTA buffer. Endogenous peroxidase activity was blocked with 3% H_2_O_2_, and non-specific binding was blocked with 5% bovine serum albumin (BSA). Sections were then incubated overnight at 4 °C with the primary antibody, and subsequently with HRP-conjugated secondary antibodies for 30 min. Staining was developed using DAB, counterstained with hematoxylin, and visualized under a microscope. The area of positive staining was quantified using ImageJ analysis software.

### RT-qPCR

Total RNA was extracted from lung tissues using the Animal Total RNA Isolation Kit (Fucheng Bio, Catalog No.: R.230701). The M gene of H1N1, as well as the genes of pro-inflammatory factors and core targets (*Myd88* and *Ccl5*) were amplified using a Real-Time PCR Detection Kit (Sibay, Catalog No.: G3337). Gene expression levels was normalized to α-Tubulin and quantified using the 2^⁻ΔΔCT^ method [18]. The primer sequences are detailed in Supplementary data 1.

### Target prediction and analysis of FQ-01

To identify the active components and their targets of FQ-01, we extracted relevant data from the TCMSP databases, applying selection criteria where oral bioavailability ≥ 30% and drug likeness ≥ 0.18. Subsequently, we obtained the disease targets for “influenza A virus” from the GeneCards database and matched them with the targets of the herbal components to identify the potential targets of FQ-01 for influenza treatment. Using the STRING database, we performed interaction analysis on these matched targets to construct a protein–protein interaction (PPI) network. We then imported this PPI network into Cytoscape 3.10.2 software and calculated the network’s topological properties using the “cytoHubba” plugin. Based on the criteria of betweenness centrality greater than the median, closeness centrality greater than the median, and degree greater than three times the median, we ranked the targets and ultimately identified the hub targets. Subsequently, we conducted KEGG pathway analysis and GO functional annotation analysis on these hub targets using the DAVID database. Finally, we constructed a disease-drug-component-target network for FQ-01 using Cytoscape 3.10.2 software.

### RNA sequencing data acquisition

The original dataset of H1N1-infected model and control groups (GSE163959) was obtained from the GEO database. Subsequently, this dataset was analyzed for differential genes using the DESeq2 software package of the Microbiotics platform (https://www.bioinformatics.com.cn/), and the differential expressed genes (DEGs) were identified by the criteria of *p*-value < 0.05 and |log (FC)|> 0. Finally, the DEGs were visualized by volcano diagrams.

### Weighted gene coexpression network analysis (WGCNA)

To identify genes that are highly associated with IAV infection, we employed the WGCNA package to construct a scale-free coexpression network using the DEGs from the GSE163959 dataset. We determined an appropriate soft-thresholding power through power-law fitting, subsequently calculated the Pearson correlation between gene expression values, and constructed a module eigengene matrix. Ultimately, identified the feature module genes that are most significantly associated with IAV infection.

### Machine learning (LASSO regression analysis)

To pinpoint potential candidate targets of FQ-01 for IAV therapy, we intersected the feature module genes previously identified with the predicted hub targets. Subsequently, we performed LASSO regression analysis on these intersected genes using the “glmnet” R package, ultimately determining the potential targets for FQ-01 in IAV treatment.

### Transcriptomic analysis

Lung tissues were collected from mice (n = 6) for subsequent transcriptomic analysis. Total RNA extraction and subsequent procedures were conducted by Novogene (Beijing Genomics Institute, Beijing, China). DEGs were identified using the criteria of *p*-value < 0.05 and |log (FC)|> 0. KEGG pathway analyses were conducted on the transcriptomic data via the R package clusterProfiler (Version 3.0.3), as provided by the Novogene online platform (https://magic.novogene.com).

### Acquisition of FQ-01 key gene

To further pinpoint the key genes of FQ-01 for IAV treatment, we intersected the genes identified through Lasso regression algorithm with those DEGs derived from the mouse lung tissue transcriptome.

### Molecular docking

The core active ingredients obtained from the “disease-drug-component-target” network were molecularly docked with the key genes. The structures of the key genes and the active components of FQ-01 were obtained from the PDB and PubChem databases, respectively. Using AutoDock Tools 4.2 software, hydrogen atoms were added to the targets and active components, charges were assigned, and atom types were labeled, generating receptor files in PDBQT format. Subsequently, we then carried out molecular docking using AutoDock Vina 1.2, and analyzed the results with Pymol 2.6.

### Molecular dynamics (MD) Simulation

We used the pdb2gmx to construct the topology of the protein with the CHARMM36 force field and TIP3P water model. Subsequently, we added hydrogen atoms to the active components and corrected them to generate the corresponding topology files in Avogadro software. Next, we integrated the structures and topology files of the protein and ligand, defined the simulation box, solvated the system with water, and added ions. The system underwent two equilibration stages: 100 picoseconds of NVT equilibration at 298 K, followed by 100 picoseconds of NPT equilibration at 1 bar. Afterward, a 100-ns MD simulation was performed. Finally, the root-mean-square deviation (RMSD) was calculated and visualized.

### Untargeted metabolomics

Lung Tissue Metabolite Extraction: Lung tissues were homogenized in a pre-chilled methanol–water solution (4:1, v/v), incubated at − 80 °C for 4 h, and then centrifuged at 14,000 rpm for 10 min at 4 °C. The supernatant was evaporated and reconstituted in 30 µL of internal standard solution (500 ng/mL dexamethasone in 80% methanol). Samples were centrifuged at 16,500 rpm for 8 min at 4 °C, and 20 µL of supernatant was used for analysis.

Serum Metabolite Extraction: Serum samples (80 µL) were mixed with 400 µL of pre-chilled methanol, vortexed, and incubated for 30 min. After centrifugation at 16,500 rpm for 5 min, the supernatant was dried and reconstituted in 30 µL of internal standard solution. A 20 µL aliquot was used for mass spectrometry analysis.

Mass Spectrometry Conditions: Metabolomic profiling was performed using a Q Exactive mass spectrometer with an Acquity BEH C18 column (2.1 mm × 100 mm, 1.7 µm). Metabolite were analyzed using MZmine 3.0, the differential metabolite (DM) were identified using the criteria of *p* < 0.1 and |log (FC)|> 0, and pathway enrichment was performed using the MetaboAnalyst platform (https://new.metaboanalyst.ca).

### Correlation analysis of metabolomics

To identify the key metabolites of FQ-01 in the treatment of IAV, we employed the “corrplot” from the R package to screen for DMs that are significantly correlated with core genes. We defined significance with criteria of an absolute correlation coefficient greater than 0.5 and a *p*-value less than 0.05. The correlations were visualized using a heatmap.

### Statistical Analysis

Data were analyzed using GraphPad Prism 8 and expressed as mean ± SEM. Multiple group comparisons, one-way ANOVA was employed. Student’s t-tests were used to compare the vehicle and IAV groups.

## Results

### FQ-01 reduced mortality and morbidity after IAV infection in mice

**Fig. 1:**
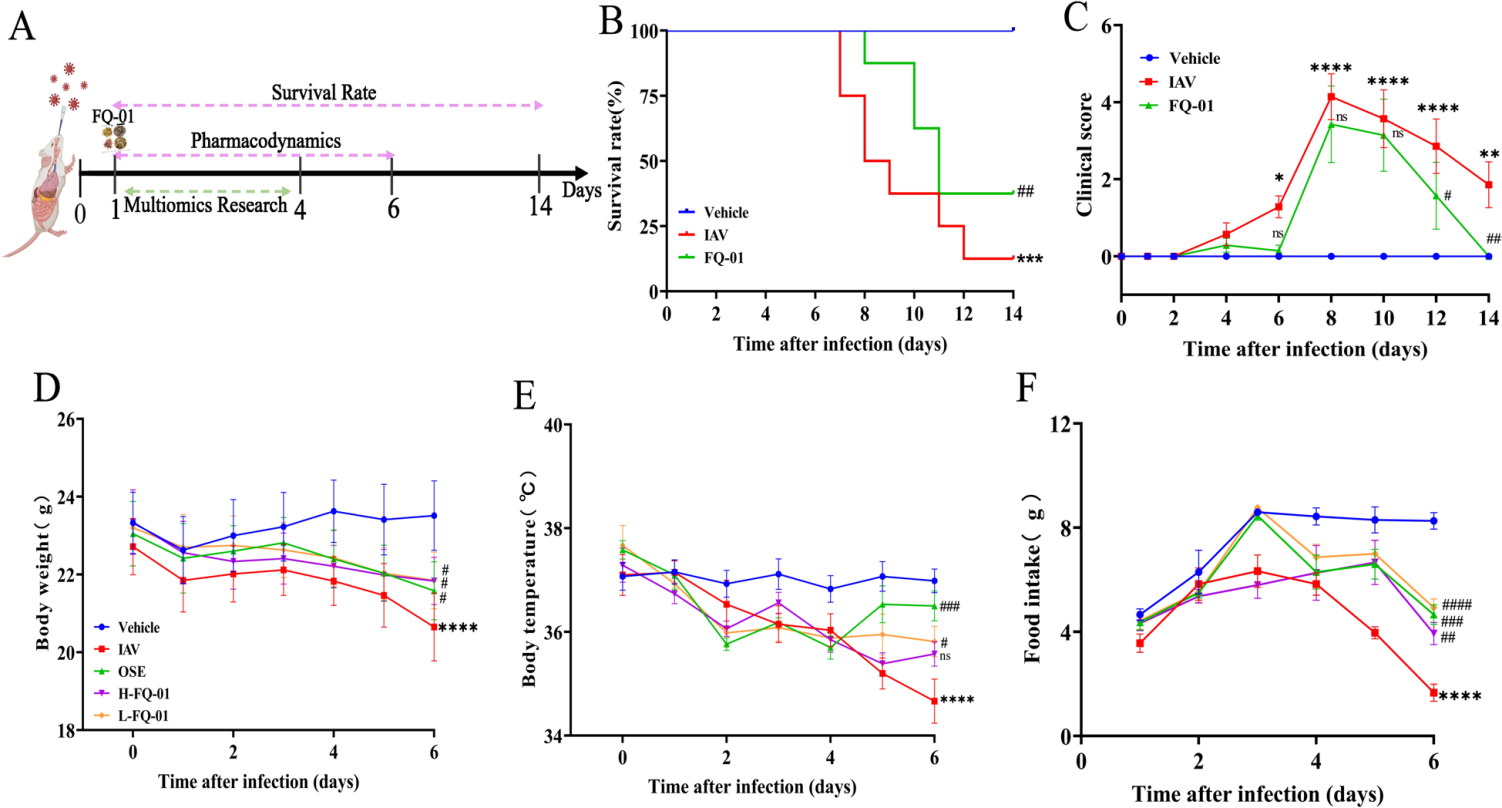
The protective effect of FQ-01 on the mortality and morbidity of mice infected with IAV. **A** Flow chart of animal experiment. **B** Effect of FQ-01 on the survival rate of mice infected with influenza (n = 8 per group). **C** Impact of FQ-01 on clinical symptom scores in influenza-infected mice (n = 7–8). **D** Mouse body weight dynamics in different groups after IAV attack (n = 6–8). **E** Changes in body temperature of mice in each group after IAV attack (n = 6–8) **F** Changes in dietary consumption of mice in each group after IAV attack (n = 6–8). All Data are presented as mean ± SEM. Significance levels: ^*^p < 0.05, ^**^p < 0.01 (vs. Vehicle group); ^#^*p* < 0.05, ^##^*p* < 0.01 (vs. IAV group)

To evaluate the protective effects of FQ-01 against IAV infection, Balb/c mice were challenged with both lethal (0.8 LD₅₀) and sub-lethal (0.4 LD₅₀) doses of IAV (Fig. 1A). In the lethal dose model, FQ-01 treatment significantly prolonged survival and reduced mortality compared to the IAV group (*p* < 0.01) (Fig. 1B). Clinical symptoms such as ruffled fur, hunched posture, and reduced activity emerged 3–4 days post-infection, with symptom severity peaking on day 8. FQ-01 treatment Markedly alleviated clinical symptom scores, leading to near-complete recovery by day 14. In contrast, the IAV group exhibited persistently elevated scores (*p* < 0.01) (Fig. 1C). Moreover, both H-FQ-01 and L-FQ-01 treatments effectively mitigated weight loss, normalized body temperature, and improved appetite in infected mice (Fig. 1D–F). Notably, L-FQ-01 outperformed OSE in restoring body weight and temperature. Consistently, at a sub-lethal dose model, we observed similar protective effects of FQ-01 on mice (Supplementary Fig. 2A and B). These findings demonstrate that FQ-01 significantly reduces morbidity and mortality in IAV-infected mice.

### FQ-01 inhibits viral replication and inflammation in mouse lungs

**Fig. 2:**
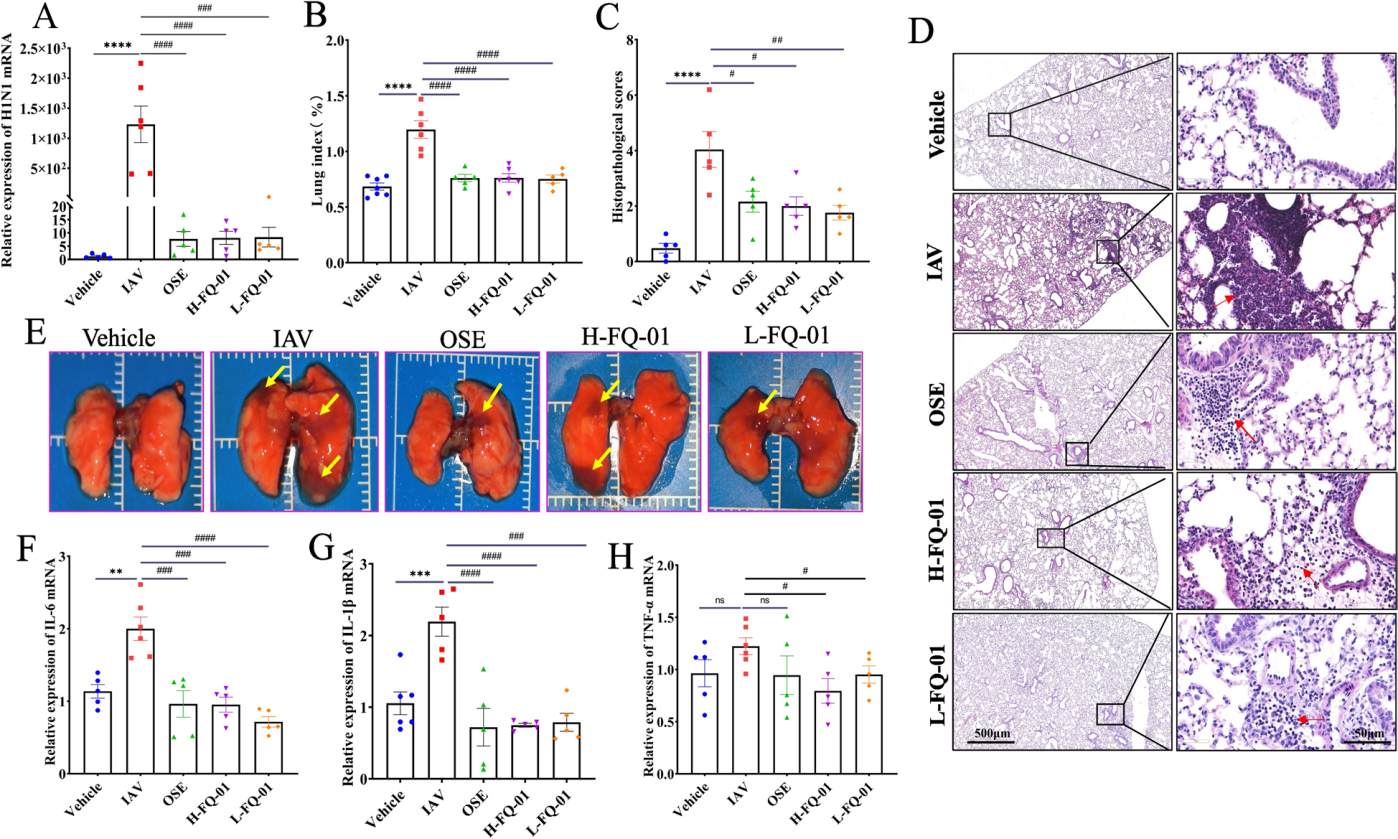
Inhibitory effects of FQ-01 on viral replication and lung inflammation in IAV-infected mice. **A** RT-PCR was used to analyze the changes of lung viral load in mice infected with IAV. **B** Changes of lung index of mice infected with virus in each group. **C** Changes of pathological score in mice. **D** Representative H&E-stained lung sections from each group (red arrows indicate inflammatory cell infiltration; bar = 50 μm). **E** Anatomy of lung tissues in each group (yellow arrows indicate areas of pulmonary congestion and consolidation). **F-H** mRNA expression levels of inflammatory cytokines in lung tissues, including IL-6 (**F**), IL-1β (**G**), and TNF-α (**H**). All data are summarized as mean ± SEM. Significance levels: ^*^p < 0.05, ^**^p < 0.01 (vs. Vehicle group); ^#^*p* < 0.05, ^##^*p* < 0.01 (vs. IAV group)

To further assess the antiviral efficacy of FQ-01, viral load in lung tissues was quantified using RT-qPCR. FQ-01 treatment significantly suppressed viral replication (Fig. 2A and Supplementary Fig. 2C). Additionally, the lung index, which serves as an indicator of pulmonary inflammation, was markedly reduced in FQ-01-treated mice compared to the IAV group (Fig. 2B and Supplementary Fig. 2D). Histopathological analysis showed that IAV infection caused extensive structural damage to alveoli, interstitium, alveolar ducts, and bronchioles, accompanied by significant lymphocytic infiltration (Fig. 2D and Supplementary Fig. 2E, red arrows). Both FQ-01 and OSE treatments improved pulmonary pathology and reduced inflammation scores, with L-FQ-01 showing particularly pronounced effects (Fig. 2C and Supplementary Fig. 2F). Gross examination of lung tissues revealed that FQ-01 alleviated lung congestion and edema (Fig. 2E). RT-qPCR analysis further demonstrated that FQ-01 significantly downregulated the expression of pro-inflammatory cytokines, including IL-6, IL-1β, and TNF-α in lung tissues (Fig. 2F–H and Supplementary Fig. 1G, H, I). Collectively, these results indicate the dual role of FQ-01 in suppressing viral replication and alleviating pulmonary inflammation, underscoring its potential as an anti-influenza therapeutic agent.

### Hub targets and components of FQ-01 for the treatment of IAV

**Fig. 3:**
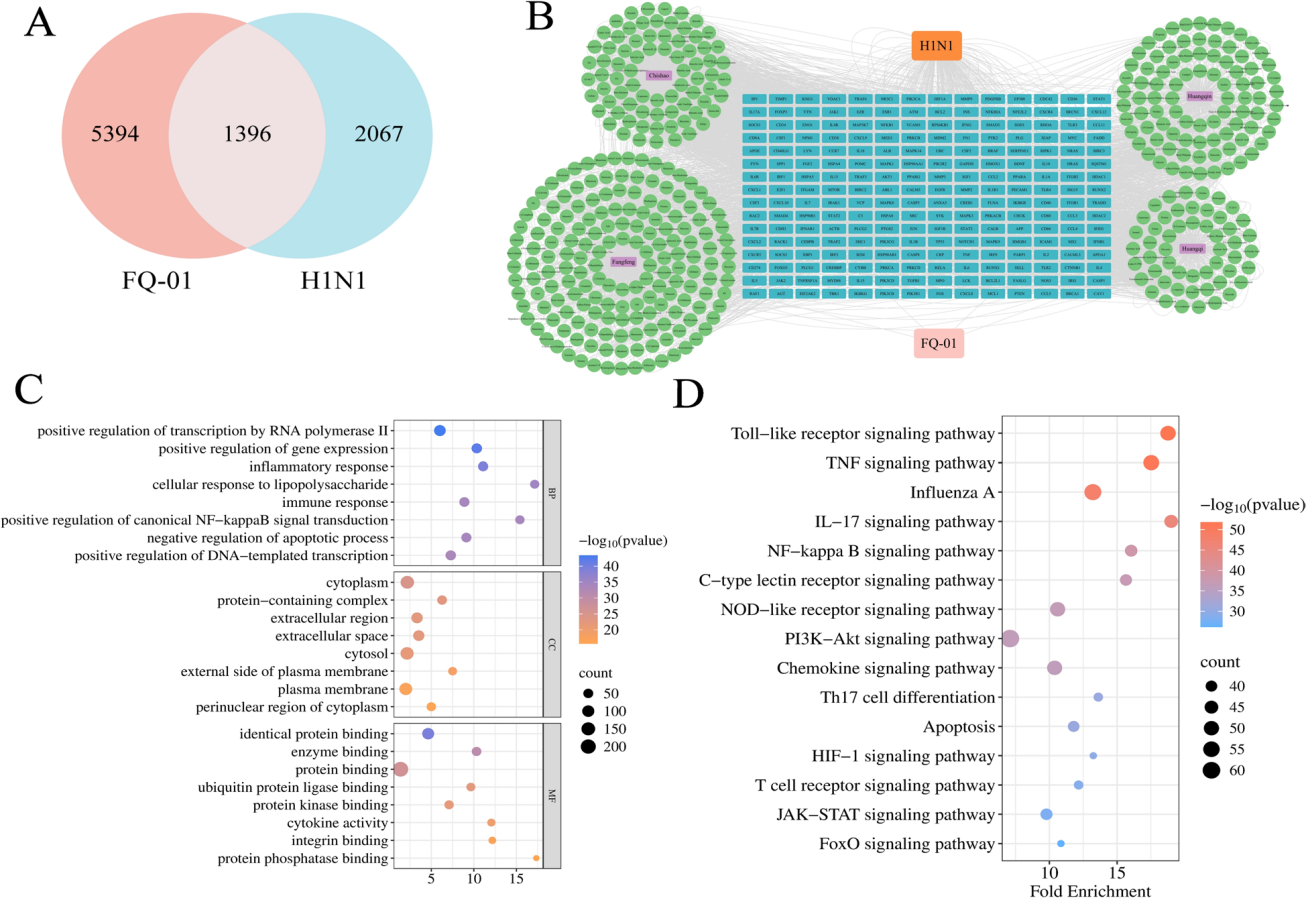
Network pharmacology prediction results of FQ-01 treatment for IAV. **A** Venn diagram showing the overlap between FQ-01 treatment targets and H1N1 disease targets. **B** Network interaction diagram of “drug-disease-component-target” for FQ-01 in treating H1N1. **C** Bubble chart of GO functional enrichment analysis for the hub targets of FQ-01. **D** Bubble chart of KEGG pathway enrichment analysis for the hub targets of FQ-01

After eliminating duplicates, we collected a total of 430 active components and 6790 targets from the TCMSP databases. Additionally, 3463 targets associated with IAV were obtained from the GeneCards database. By intersecting the targets of the active components with the disease-related targets, we identified 1396 potential targets for FQ-01 in the treatment of IAV (Fig. 3A). Further PPI analysis led to the selection of 224 core targets, and a network of 234 hub targets and their corresponding active components was constructed using Cytoscape (Fig. 3B). GO enrichment analysis and KEGG pathway analysis were performed to elucidate the biological significance of these core targets. The GO analysis revealed that these targets are mainly involved in biological processes (BP) like “positive regulation of gene expression,” “inflammatory response,” and “immune response,” as well as molecular functions (MF) like “identical protein binding” and “enzyme binding” (Fig. 3C). KEGG Pathway enrichment further revealed that these core targets are closely related to innate immune signaling pathways (e.g., Toll-like receptor, Th17, and NOD-like recepter) and inflammatory pathways (e.g., TNF, NF-κB, and JAK-STAT) (Fig. 3D).

### Identification of potential gene for FQ-01 in treating IAV based on WGCNA and machine learning algorithms

**Fig. 4:**
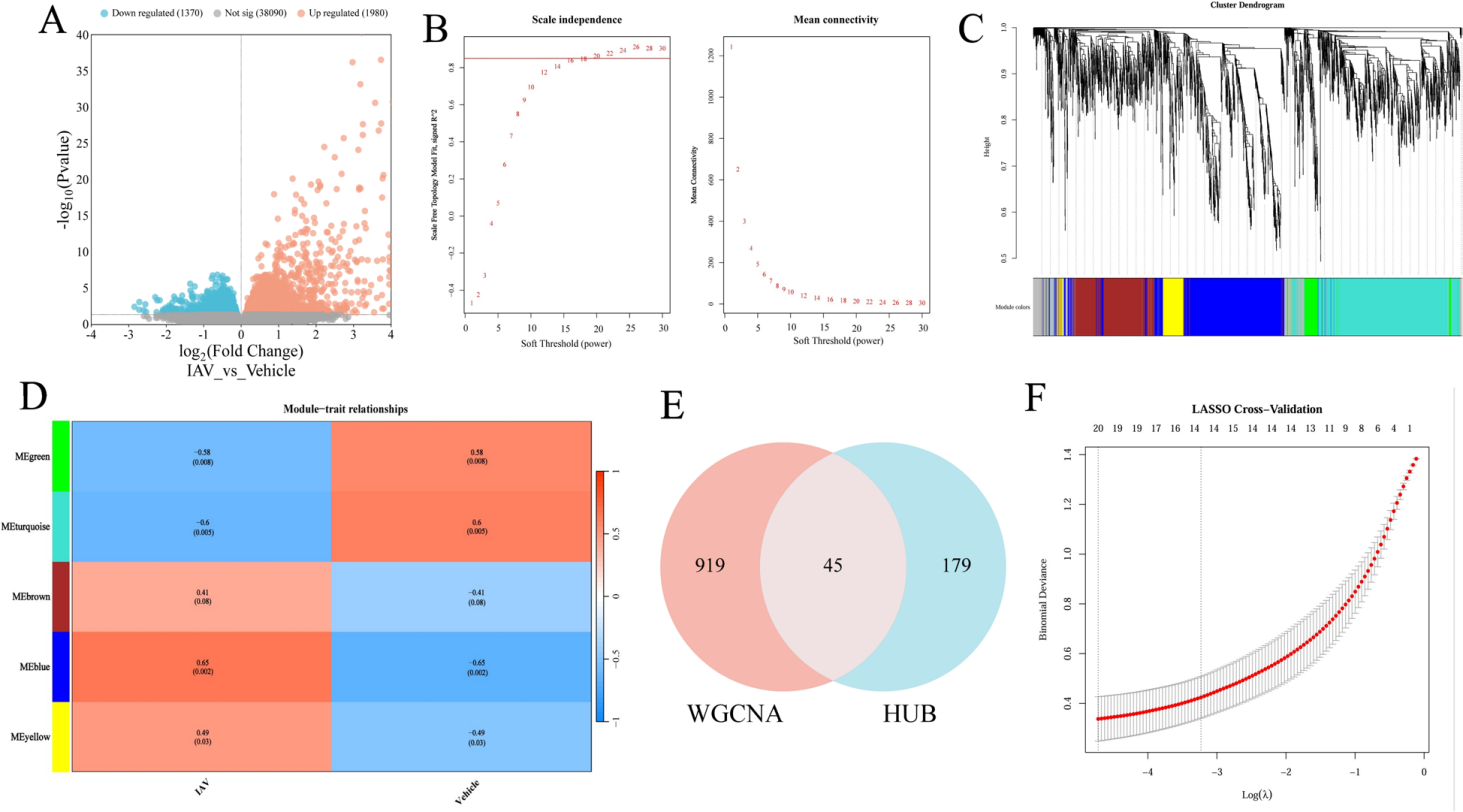
Differential gene analysis, WGCNA, and machine learning-based prediction of FQ-01 targets using the GSE163959 dataset. **A** Volcano plot of DEGs between H1N1 and control groups in the GSE163959 dataset. **B** Network plot for soft-threshold selection in WGCNA analysis of DEGs. **C** Hierarchical clustering dendrogram of DEGs based on similar expression patterns. **D** Correlation plot between gene modules and H1N1 infection. **E** Venn diagram showing the intersection of highly correlated module genes from WGCNA analysis and hub genes predicted by network pharmacology. **F** Lasso regression analysis of the 45 intersecting genes to determine the minimum number of diagnostic biomarkers

In the GSE163959 dataset, differential expression analysis revealed 3350 DEGs between the H1N1 infection group and the vehicle group, comprising 1980 upregulated and 1370 downregulated genes (Fig. 4A). Subsequently, WGCNA was performed on these DEGs. In the WGCNA analysis, samples were clustered based on Pearson correlation coefficients, and the optimal soft threshold was determined to be 16 (Fig. 4B). Using this threshold, five modules were generated (Fig. 4C), among which the blue module (964 genes, r = 0.65, *p *= 0.002) exhibited the strongest association with H1N1 infection (Fig. 4D). Further intersection analysis identified 45 potential targets for FQ-01 in the treatment of IAV among the genes in the blue module and the 224 hub targets (Fig. 4E).

GO analysis of these 45 potential targets revealed that the BP were primarily enriched in “defense response to virus,” “response to lipopolysaccharide,” and “inflammatory response,” while the MF were mainly related to “protein binding” (Supplementary Fig. 3A). KEGG pathway analysis indicated that these targets were significantly associated with the “Toll-like receptor signaling pathway,” “Influenza A,” and “TNF signaling pathway” (Supplementary Fig. 3B). Finally, LASSO regression analysis was conducted on these 45 potential targets, and the results highlighted 20 genes, including *Mpo*, *Foxp3*, *Tradd*, *Stat1*, *Mx1*, *Myd88*, *Tnf*, *Ccl5*, etc., as candidate genes for FQ-01 in treating IAV (Fig. 4F).

### Transcriptomic analysis of lung tissue after IAV infection in mice

**Fig. 5:**
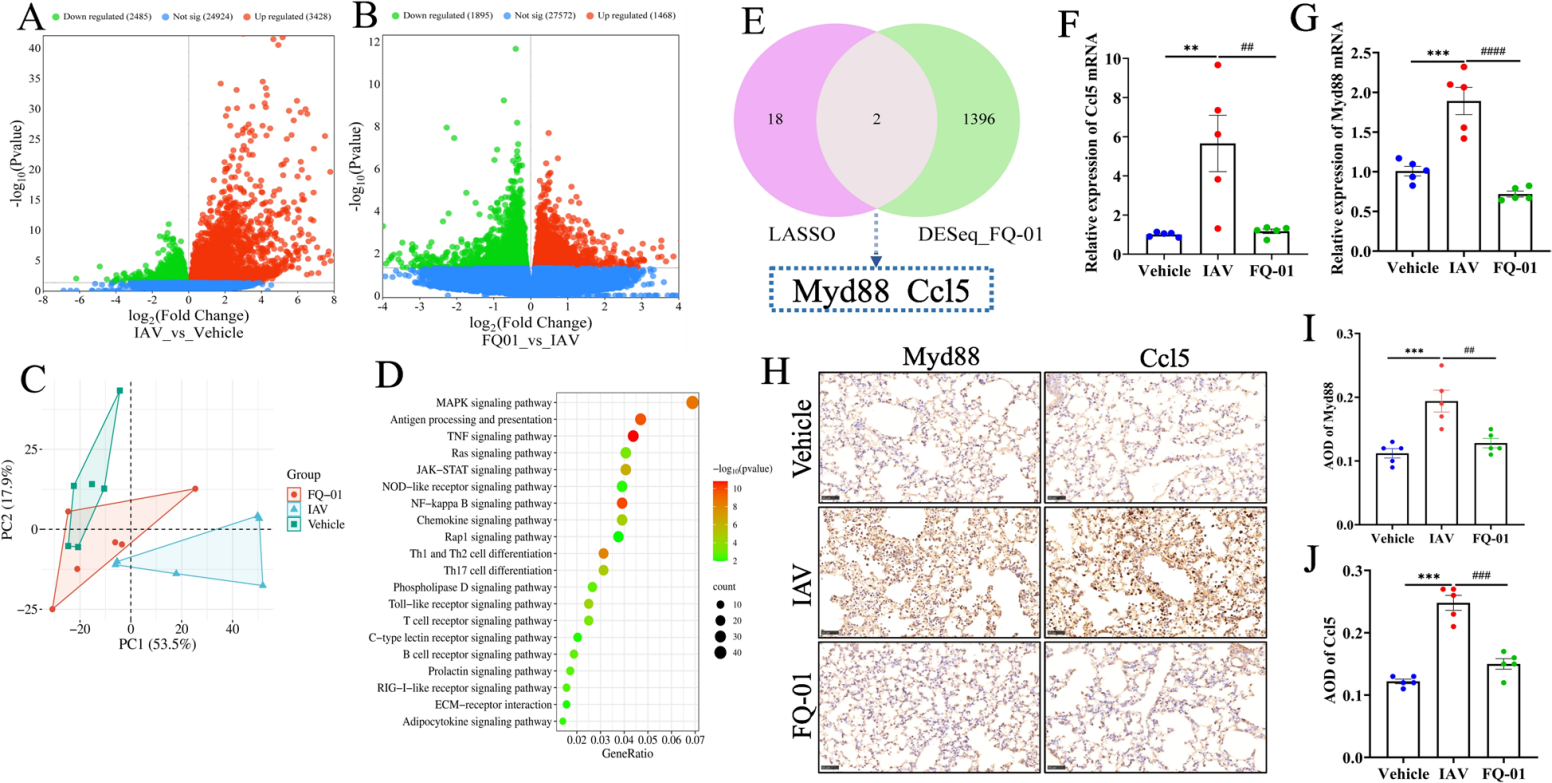
Transcriptome analysis of IAV mice treated with FQ-01. **A** DEGs volcano plot of IAV vs vehicle group in mouse lung tissue. **B** DEGs volcano plot of FQ-01 treated vs IAV group. **C** PCA diagram of DEGs effectively regulated by FQ-01. **D** KEGG pathway enrichment analysis of DEGs in mouse lung tissue. **E** Venn diagram showing the intersection of DEGs from mouse lung tissue transcriptome analysis and candidate genes from LASSO regression analysis. **F–G** RT-qPCR validation results for the key genes Myd88 and Ccl5 (n = 5). **H** Representative immunohistochemical staining images of Myd88 and Ccl5 protein levels in mouse lung tissues. **I–J** Quantitative analysis of Myd88 and Ccl5 protein expression

To elucidate the underlying mechanisms of FQ-01 in antiviral and anti-inflammatory effects, transcriptomic sequencing was performed on lung tissues. Compared to the vehicle group, the IAV group exhibited 5,913 DEGs, including 3,428 upregulated and 2,485 downregulated genes (Fig. 5A). FQ-01 treatment reversed these changes, with 3,363 DEGs identified between the FQ-01 and IAV groups (1,468 upregulated and 1,895 downregulated) (Fig. 5B). Venn diagram analysis revealed 1,498 DEGs associated with FQ-01’s therapeutic effects. Of these, 377 genes were downregulated during infection but upregulated following FQ-01 treatment, while 1,021 genes were upregulated during infection but downregulated after treatment with FQ-01 (Supplementary Fig. 3C and D). Principal component analysis (PCA) further confirmed the robustness of the transcriptomic data (Fig. 5C). KEGG pathway enrichment analysis of the 1,498 DEGs highlighted several key pathways involved in inflammation, such as the TNF, NF-κB, MAPK, and JAK-STAT signaling pathways, as well as pathways related to innate immunity, including Toll-like receptor signaling and Th17 cell differentiation (Fig. 5D). Two key genes, *Myd88* and *Ccl5*, resulted from the intersection of 1498 DEGs with Lasso-predicted candidate genes (Fig. 5E). Both RT-qPCR (Fig. 5F and G) and immunohistochemistry (Fig. 5H, I and J) analyses demonstrate that FQ-01 significantly suppresses the increased expression of Myd88 and Ccl5 at both the mRNA and protein levels induced by IAV infection.

### Metabolomics analysis of lung tissue and serum in mice infected with IAV

**Fig. 6:**
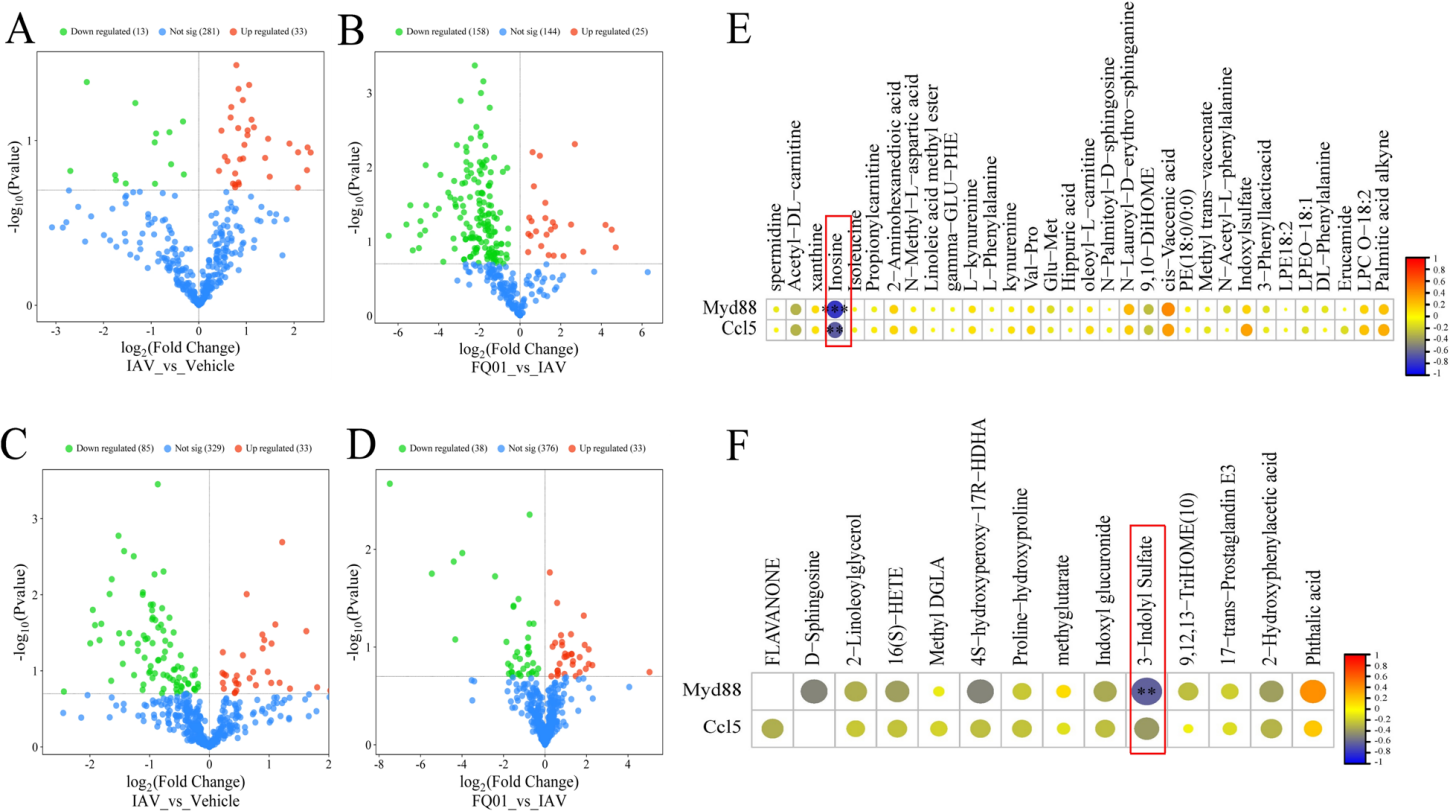
Metabonomic analysis of lung and serum in IAV mice treated with FQ-01. **A** Volcano plot of DMs in lung tissue between IAV and vehicle groups. **B** Volcano plot of DMs in lung tissue between FQ-01 and IAV groups. **C** Volcano plot of DMs in serum between IAV and vehicle group. **D** Volcano plot of DMs in serum between FQ-01 and IAV groups. **E** Pearson correlation thermogram between myd88, ccl5 and lung DMs. **F** Pearson correlation thermogram between myd88, ccl5 and serum DMs

In the lung tissues, the IAV group exhibited 46 differential metabolites (DMs), including 33 upregulated and 13 downregulated metabolites compared to the vehicle group (Fig. 6A). FQ-01 treatment reversed these changes, with 183 DMs identified, including 25 upregulated and 158 downregulated metabolites (Fig. 6B). In serum, the IAV group exhibited 118 DMs compared to the vehicle group (33 upregulated and 85 downregulated) in serum (Fig. 6C). Following FQ-01 treatment, 71 DMs were identified, with 33 upregulated and 38 downregulated metabolites (Fig. 6D). Venn diagram analysis revealed 32 DMs in the lung and 14 DMs in the serum associated with FQ-01’s therapeutic effects (Supplementary Figs. 4A, 4B, 4C and 4D), and the heatmap illustrated the reversal of metabolite levels following FQ-01 treatment (Supplementary Fig. 4E and F). Pearson correlation analysis was conducted between the key genes *Myd88* and *Ccl5*, and the DMs in lung tissue and serum. The results indicated that in lung tissue, inosine was significantly negatively associations with *Myd88* (r = − 0.77, *p* = 0.0002) and *Ccl5* (r = − 0.62, *p* = 0.006) (Fig. 6E). Additionally, in serum, the metabolite 3-indoly sulfate exhibited a notable negative correlation with *Myd88* (r = − 0.60, *p* = 0.008) (Fig. 6F).

### Molecular docking and MD simulation verification

**Fig. 7:**
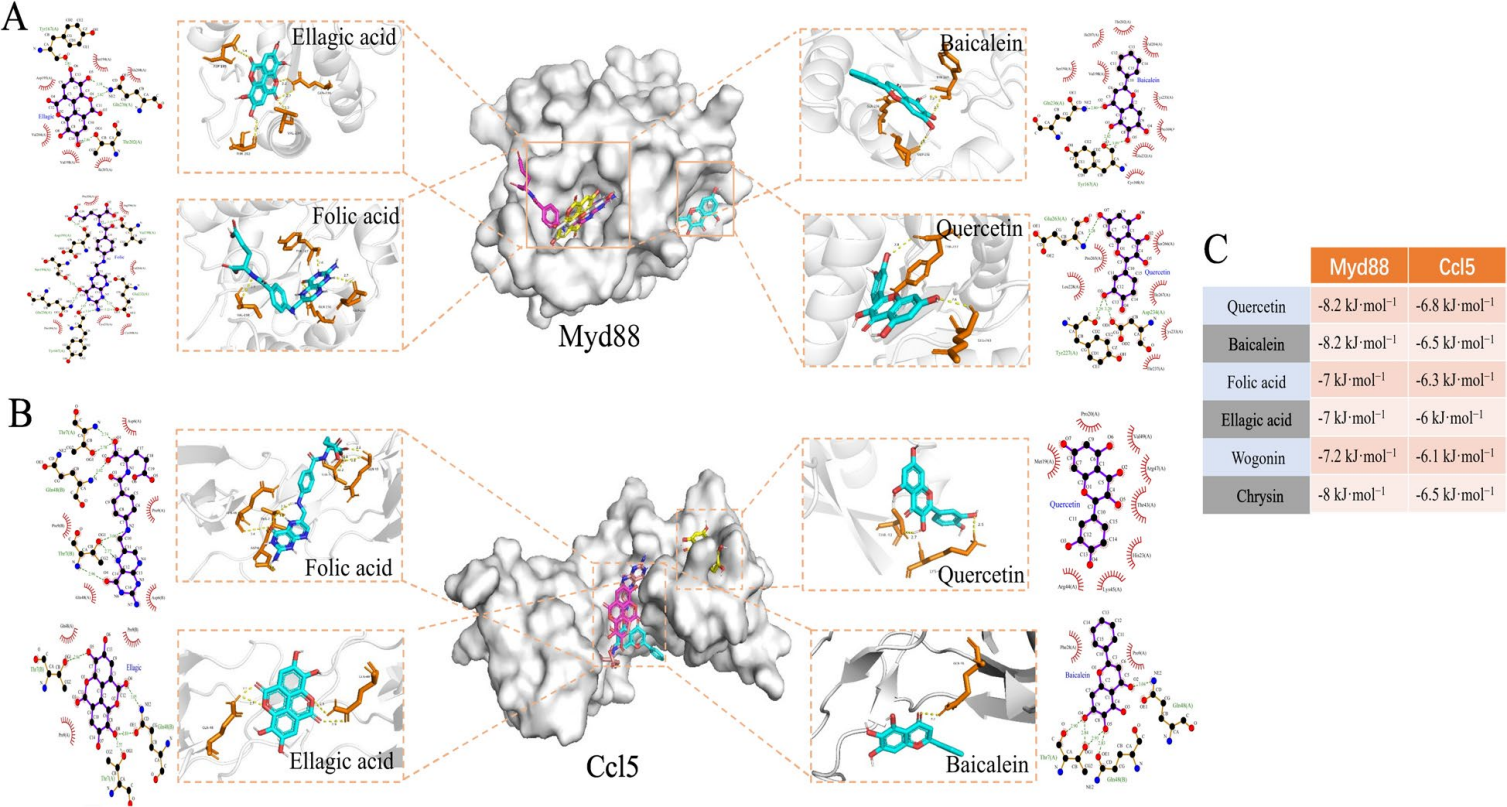
Molecular docking. **A** Visualization results of molecular docking between four core active components (Ellagic acid, Folic acid, Baicalein, Quercetin) in FQ-01 and Myd88. **B** Visualization results of molecular docking between four core active components (Ellagic acid, Folic acid, Baicalein, Quercetin) in FQ-01 and Ccl5. **C** Docking binding energies between the top six core active components in FQ-01 and Myd88 and Ccl5

Molecular docking was performed between the six hub active components of FQ-01 and the *Myd88* and *Ccl5* targets. The results showed that the binding energies of all active components with the targets were lower than − 6 kcal/mol (Fig. 7C). The key interactions between *Myd88* and *Ccl5* with four active components were illustrated by LigPlus 2.2 (2D) and Pymol 2.5 (3D) (Fig. 7A, 7B). Specifically, the binding energies of Myd88 with baicalein and quercetin were − 8.2 kcal/mol (Fig. 7C).

**Fig. 8:**
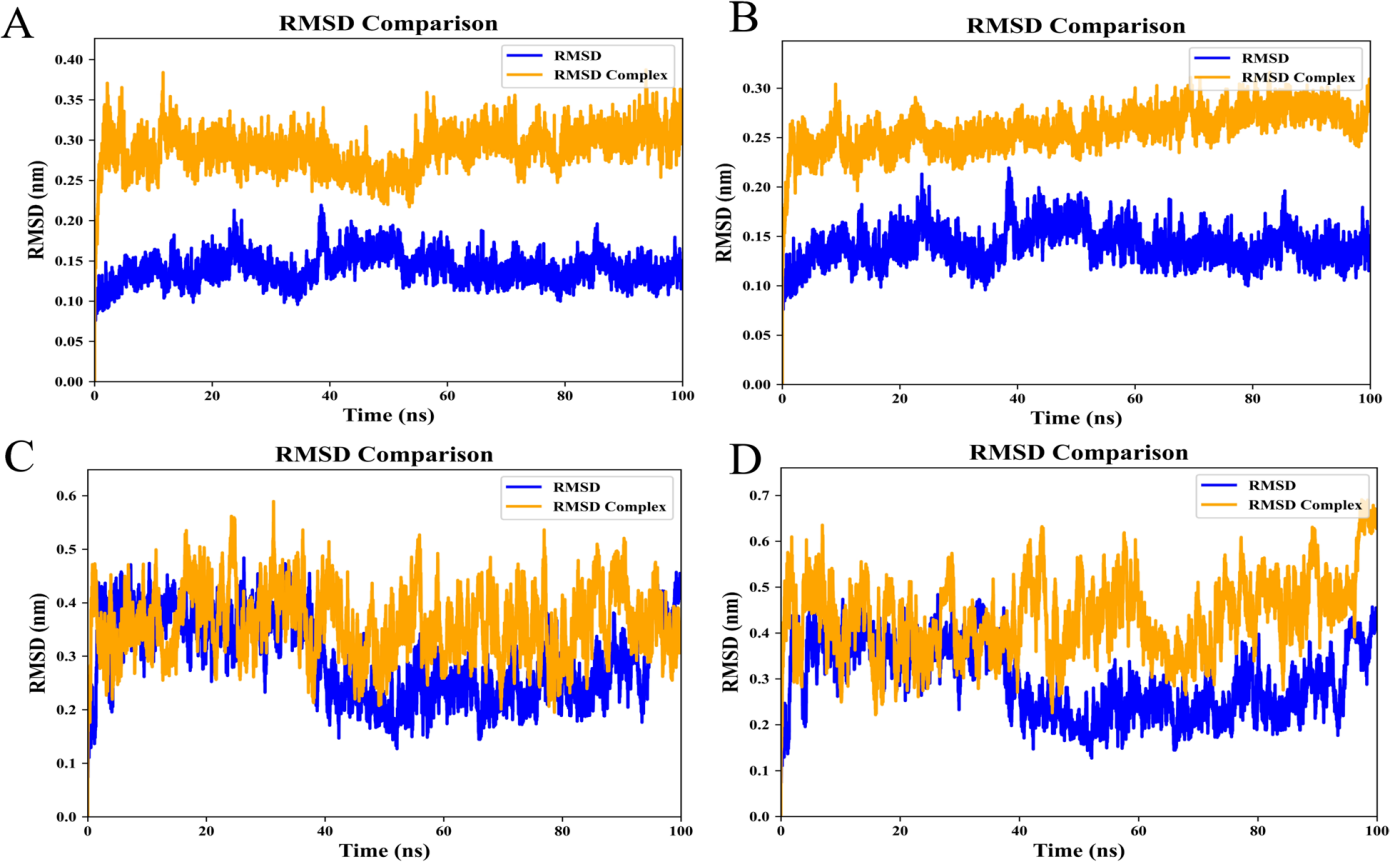
Molecular dynamics simulation: The RMSD values of the molecular complex system within a time interval of 100 ns. **A** The dynamic stability of the Myd88 protein (blue line) and the baicalein complex (orange line). **B** The dynamic stability of the Myd88 protein (blue line) and the quercetin complex (orange line). **C** The dynamic stability of the Ccl5 protein (blue line) and the baicalein complex (orange line). **D** The dynamic stability of the Ccl5 protein (blue line) and the quercetin complex (orange line)

MD simulations further confirmed the binding stability between the proteins and ligands. The RMSD values, reflecting the similarity of molecular conformations, indicate greater stability of the structures with lower RMSD values [19]. The simulation results showed that the RMSD values of *Myd88* and *Ccl5* proteins fluctuated in sync with those of their respective complexes and remained relatively stable within 100 ns, demonstrating stable binding interactions between *Myd88* and *Ccl5* and the active components of FQ-01, baicalin and quercetin (Fig. 8A, B, C and D).

## Discussion

The pathogenesis of IAV infection is characterized by two critical factors: high viral replication and excessive inflammatory responses, both of which contribute significantly to pulmonary impairment [20–22]. Our experimental data demonstrated that FQ-01 treatment conferred substantial protection in IAV-infected mice, as evidenced by improved survival rates, reduced clinical symptom scores, suppressed viral load and attenuated expression of pro-inflammatory cytokines. Bioinformatics analysis combined with database mining has been widely used for target prediction of TCM in the treatment of human diseases [23, 24]. To systematically investigate FQ-01’s mechanisms, we employed an integrated computational approach. Network pharmacology analysis combined with WGCNA of H1N1-infected lung tissue GEO datasets identified 45 putative therapeutic targets (Fig. 4E). GO functional enrichment and KEGG pathway analyses revealed that these targets were significantly enriched in viral immune responses pathways and inflammatory regulation of inflammatory processes (Supplementary Fig. 3A and B). Machine learning, which has provided more reliable support for TCM data [25]. Building on these findings, we applied Machine learning approaches to enhance target prediction reliability. LASSO regression analysis of the 45 candidate targets yielded 20 high-probability targets, including *Mpo*, *Foxp3*, *Tradd*, *Stat1*, *Mx1*, *Myd88*, *Tnf*, and *Ccl5*. Transcriptomic profiling of FQ-01-treated lungs validated these predictions, identifying 1,498 DEGs that were similarly enriched in innate immunity (e.g., Toll-like receptor, Th17, and Th1/Th2 differentiation) and inflammatory pathways (e.g., TNF, NF-κB, MAPK, and JAK-STAT). Through integrative analysis intersecting the 1,498 DEGs with our 20 candidate targets, we identified *Myd88* and *Ccl5* as the core mediators of FQ-01’s therapeutic effects. These findings establish that FQ-01 exerts its dual antiviral and immunomodulatory actions through coordinated regulation of the Myd88-dependent signaling cascade and Ccl5-mediated inflammatory responses.

*Myd88*, a central adaptor protein in innate immunity, mediates critical antiviral responses but can drive pathological inflammation when hyperactivated, leading to an overabundance of inflammatory cytokines, including IL-1β, IL-6, IFN-γ, and TNF-α [26, 27]. Similarly, *Ccl5*, a key β-chemokine, plays a dual role in influenza infection: it initially activates antiviral pathway in epithelial cells [28], including the induction of the restriction factor SAMHD1 to suppress IAV replication [29], but its sustained upregulation promotes excessive infiltration of neutrophils, inflammatory monocytes, and NK cells, exacerbating lung immunopathology [30]. Our experimental results revealed a significant upregulation of both *Myd88* and *Ccl5* gene expression in H1N1-infected lung tissues compared to controls, RT-qPCR and IHC analysis demonstrated that FQ-01 treatment effectively normalized this dysregulation, reducing *Myd88* and *Ccl5* expression to near-baseline levels. This regulatory effect correlated with a substantial decrease in pulmonary pro-inflammatory cytokines, including IL-6, TNF-α, IL-1β in the lungs. Molecular docking can reflect the static binding affinity between targets and compounds, while MD simulations can reveal their dynamic stable interaction [31]. Molecular docking studies demonstrated high-affinity binding between FQ-01’s bioactive components and both *Myd88* and *Ccl5*, with key interactions observed at their functional domains (Fig. 7). Subsequent MD simulations revealed stable binding conformations (Fig. 8). Based on these findings, we propose that *Myd88* and *Ccl5* are key targets underlying the anti-inflammatory and antiviral effects of FQ-01.

Metabolomic profiling identified 14 serum and 32 lung metabolites significantly associated with FQ-01’s therapeutic efficacy. Spearman correlation analysis demonstrated that negative correlations between *Myd88*/*Ccl5* expression levels and both serum 3-indoly sulfate (a tryptophan metabolite) and the pulmonary inosine concentrations (Fig. 6E and F). 3-Indole sulfate, a gut microbiota-derived tryptophan catabolite, mediates immunomodulatory effects through aryl hydrocarbon receptor signaling [32]. Notably, influenza infection perturbs gut microbial ecology, leading to reduced systemic 3-indole sulfate levels. Our findings suggest that FQ-01 May restore antiviral immunity by normalizing tryptophan metabolic pathways to elevate 3-indole sulfate production. Similarly, inosine, an endogenous purine nucleoside, exerts dual anti-inflammatory and antiviral properties via TLR7/8-mediated innate immune activation [33]. Our findings showed that inverse relationship between inosine and *Myd88* expression (Fig. 6E), suggesting IAV infection inhibits influenza-induced suppression of host RNA biosynthesis, which compromises antiviral defense mechanisms. We propose that FQ-01 enhances host resistance by modulating purine metabolic flux to increase inosine bioavailability.

This work presents an innovative multi-omics approach integrating network pharmacology, PPI analysis, WGCNA, and machine learning (LASSO regression) to systematically predict FQ-01’s potential anti-influenza targets and pathways. Subsequent in vivo transcriptome sequencing identified *Myd88* and *Ccl5* as key mediators of FQ-01’s antiviral effects. These findings were further validated through molecular docking, MD simulation, and in vivo RT-qPCR and IHC assays, confirming FQ-01’s regulatory impact on these targets. Moreover, Pearson correlation analysis uncovered associations between *Myd88*/*Ccl5* expression and serum/lung metabolomic profiles, linking transcriptional regulation to metabolic modulation. Collectively, our results provide a comprehensive, multi-layered mechanistic understanding of FQ-01’s antiviral activity-spanning transcriptional regulation, protein interaction networks, and metabolic reprogramming. These insights advance the mechanistic study of antiviral therapeutics and highlight FQ-01’s potential as a multi-target influenza treatment.

## Conclusions

FQ-01 demonstrates promising therapeutic potential against influenza virus infection, exerting dual antiviral and anti-inflammatory effects through multi-modal regulation of key immune mediators (*Myd88*, *Ccl5*) and metabolic modulators (3-indole sulfate, inosine). This target-metabolite interplay underscores FQ-01’s mechanistic uniqueness as a host-directed antiviral agent, offering a promising therapeutic strategy distinct from conventional viral-targeting approaches.

## Supplementary Information

Supplementary material 1. Quantification of active components in FQ-01 by high-resolution mass spectrometry. (A) Total ion current and product ion spectra of FQ-01 reference standards in positive ion mode, showing the retention times and mass-to-charge ratios (m/z) of each active component. (B) Total ion current and product ion spectra of each active component in FQ-01 samples in positive ion mode.

Supplementary material 2. The protective effect of FQ-01 on the morbidity of mice infected with IAV. (A) Mouse body weight dynamics in different groups after IAV attack (n = 6). (B) Changes in food intake of mice in each group after IAV attack (n = 6). (C) Expression of viral load in mouse lung tissue. (D) Changes of lung index of mice infected with virus in each group. (E) Representative H&E-stained lung sections from each group (red arrows indicate inflammatory cell infiltration; bar = 50 μm). (F) Changes of pathological score in mice. (G-I) mRNA expression levels of inflammatory cytokines in lung tissues, including IL-6 (G), IL-1β (H), and TNF-α (I). All data are summarized as mean ± SEM. Significance levels: ^*^p < 0.05, ^**^p < 0.011 (vs. Vehicle group); ^#^*p* < 0.05, ^##^*p* < 0.01, (vs. IAV group).

Supplementary material 3. Functional enrichment analysis of the 45 potential targets of FQ-01 against IAV. (A) Bubble plot of GO enrichment analysis for the 45 predicted potential targets. (B) Bubble plot of KEGG pathway enrichment analysis for the 45 predicted potential targets. (C) The DEGs venn diagram of FQ-01 positive regulation. (D) The DEGs venn diagram of FQ-01 negative regulation.

Supplementary material 4. Analysis of serum and lung tissue differential metabolites in mice. (A) The DMs venn diagram of FQ-01 positive regulation in lung tissue. (B) The DMs venn diagram of FQ-01 negative regulation in lung tissue. (C) The DMs venn diagram of FQ-01 positive regulation in serum. (D) The DMs venn diagram of FQ-01 negative regulation in serum. (E) Heatmap of DMs in lung tissue. (F) Heatmap of DMs in serum.

Supplementary material 5.

Supplementary material 6.

Supplementary material 7.

Supplementary material 8.

Supplementary material 9

## Data Availability

No datasets were generated or analysed during the current study.

## Abbreviations

IAV: Influenza A virus
TCM: Traditional chinese medicine
FQ-01: Fangqin qinggan decoction
WGCNA: Weighted gene co-expression network analysis
MD: Molecular dynamics
H&E: hematoxylin and eosin
PBS: Phosphate-buffered saline
BSA: Bovine serum albumin
H-FQ-01: High-dose FQ-01
L-FQ-01: Low-dose FQ-01
OSE: Oseltamivir phosphate
LD_50_: The median lethal dose
DEGs: Differentially expressed genes
HRMS: High-Resolution mass spectrometry
DMs: Differential metabolites
PPI: Protein-protein interaction
RMSD: The root-mean-square deviation
BP: Biological processes
MF: Molecular functions
PCA: Principal component analysis
MyD88: Myeloid differentiation primary response gene 88
Ccl5: C-C motif chemokine ligand 5
