# Supplementary figures and images for: Deciphering the antiviral mechanisms of Fangqin Qinggan decoction against influenza A virus: a multi-omics and machine learning approach
Source: Chin Med. 2025 Oct 5;20:158. doi: 10.1186/s13020-025-01211-0 (PMC12497347; doi:10.1186/s13020-025-01211-0)

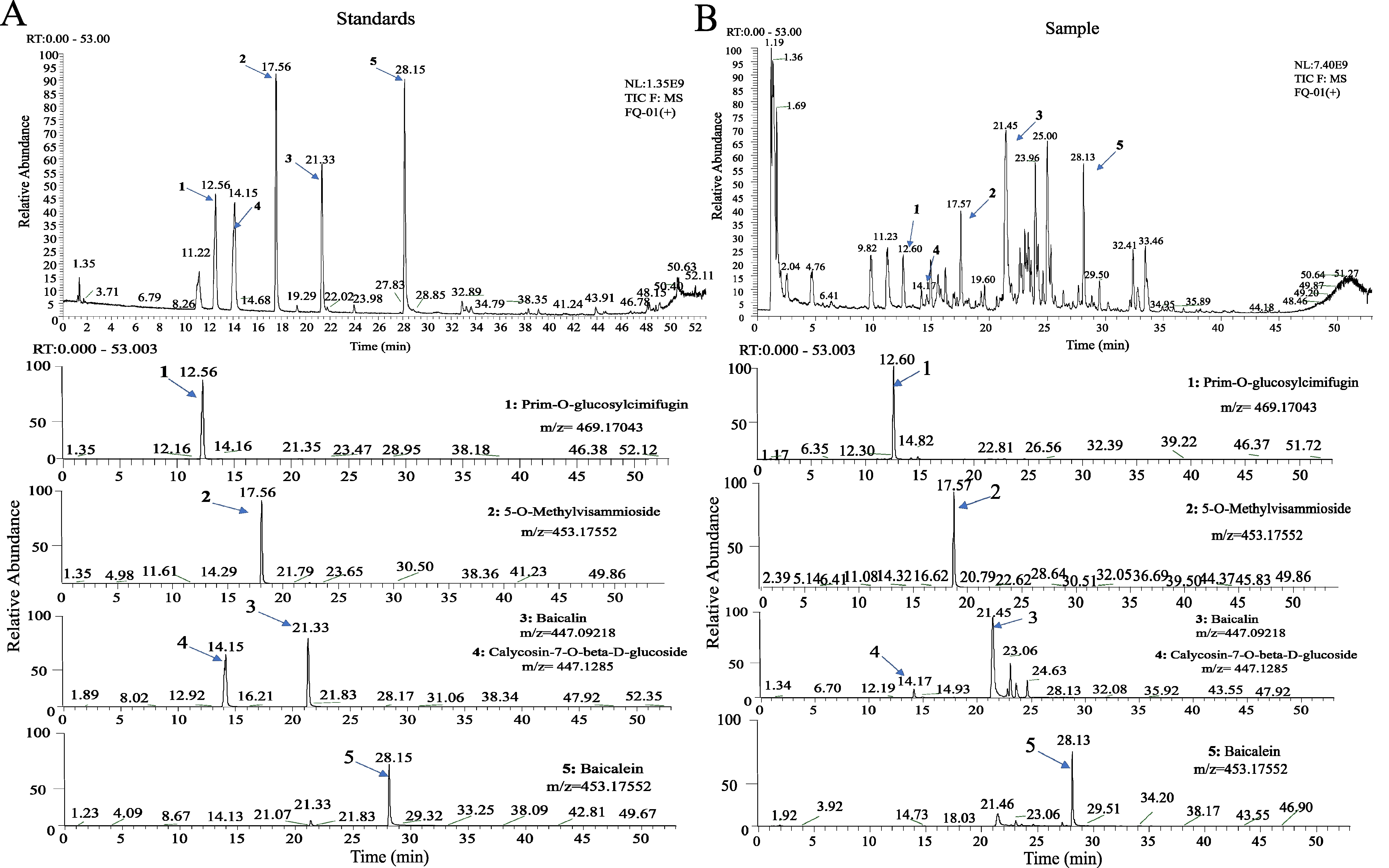

Supplement: Supplementary file 1 — Supplementary material 1. Quantification of active components in FQ-01 by high-resolution mass spectrometry. (A) Total ion current and product ion spectra of FQ-01 reference standards in positive ion mode, showing the retention times and mass-to-charge ratios (m/z) of each active component. (B) Total ion current and product ion spectra of each active component in FQ-01 samples in positive ion mode. [file 13020_2025_1211_MOESM1_ESM.jpg]

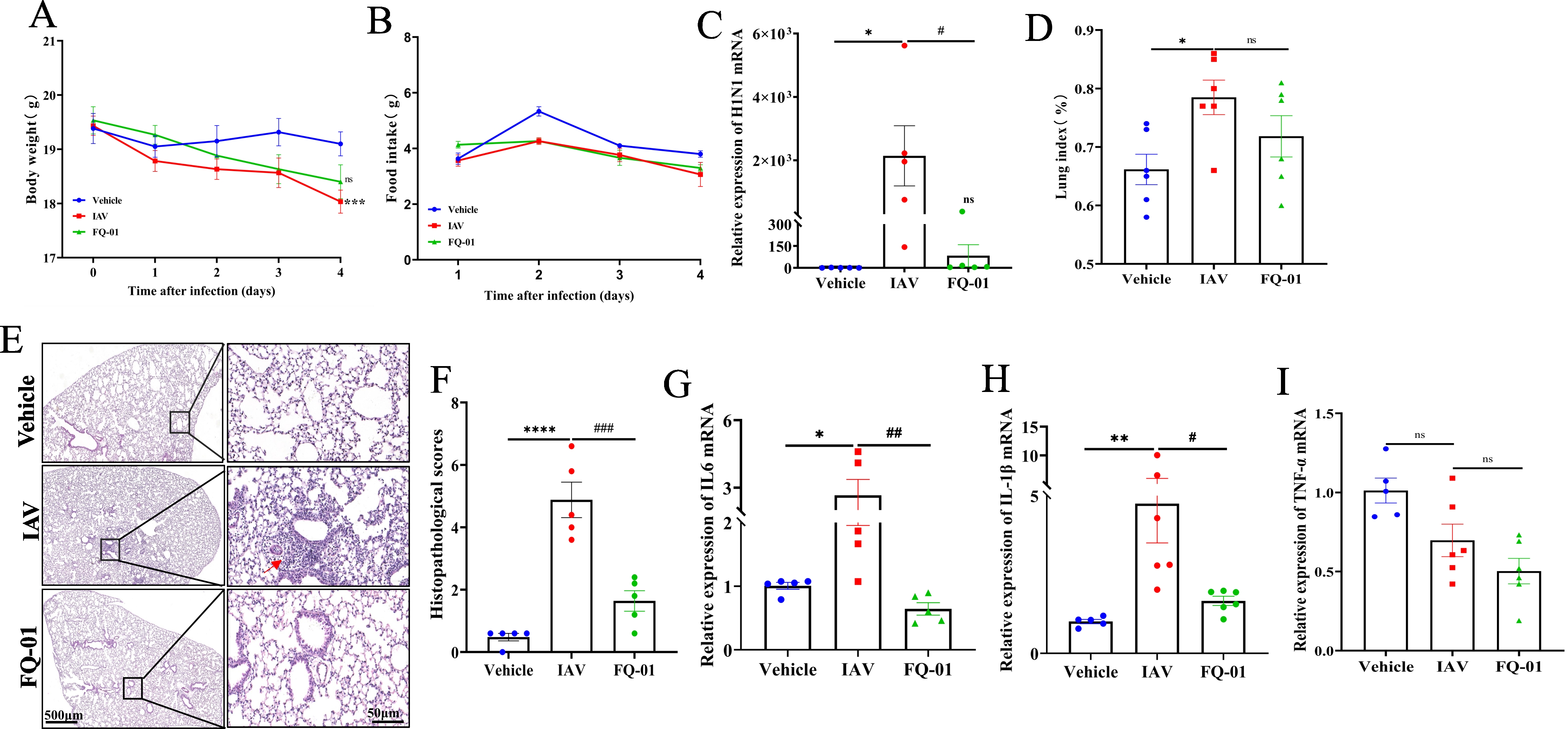

Supplement: Supplementary file 2 — Supplementary material 2. The protective effect of FQ-01 on the morbidity of mice infected with IAV. (A) Mouse body weight dynamics in different groups after IAV attack (n = 6). (B) Changes in food intake of mice in each group after IAV attack (n = 6). (C) Expression of viral load in mouse lung tissue. (D) Changes of lung index of mice infected with virus in each group. (E) Representative H&E-stained lung sections from each group (red arrows indicate inflammatory cell infiltration; bar = 50 μm). (F) Changes of pathological score in mice. (G-I) mRNA expression levels of inflammatory cytokines in lung tissues, including IL-6 (G), IL-1β (H), and TNF-α (I). All data are summarized as mean ± SEM. Significance levels: *p < 0.05, **p < 0.011 (vs. Vehicle group); #p < 0.05, ##p < 0.01, (vs. IAV group). [file 13020_2025_1211_MOESM2_ESM.jpg]

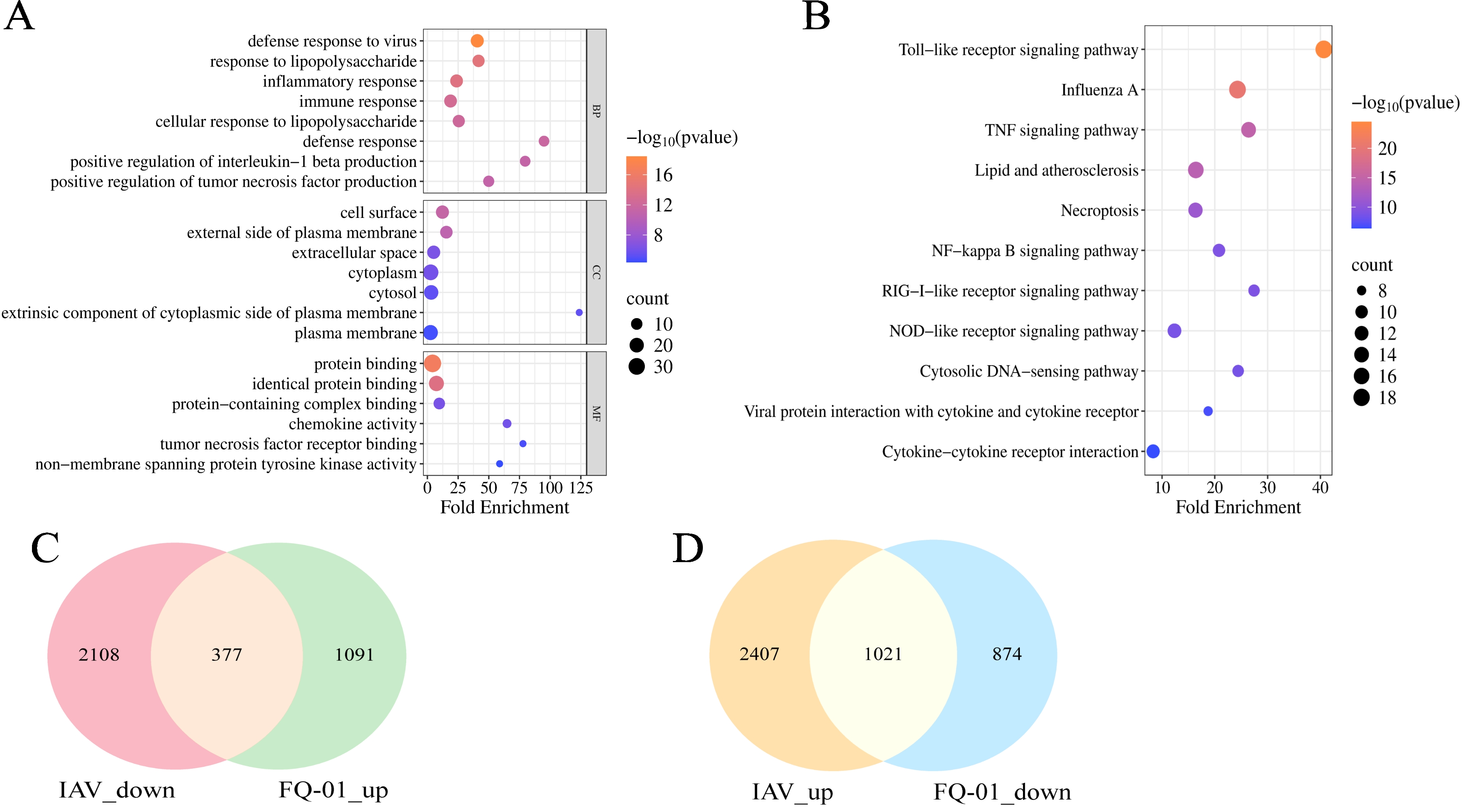

Supplement: Supplementary file 3 — Supplementary material 3. Functional enrichment analysis of the 45 potential targets of FQ-01 against IAV. (A) Bubble plot of GO enrichment analysis for the 45 predicted potential targets. (B) Bubble plot of KEGG pathway enrichment analysis for the 45 predicted potential targets. (C) The DEGs venn diagram of FQ-01 positive regulation. (D) The DEGs venn diagram of FQ-01 negative regulation. [file 13020_2025_1211_MOESM3_ESM.jpg]

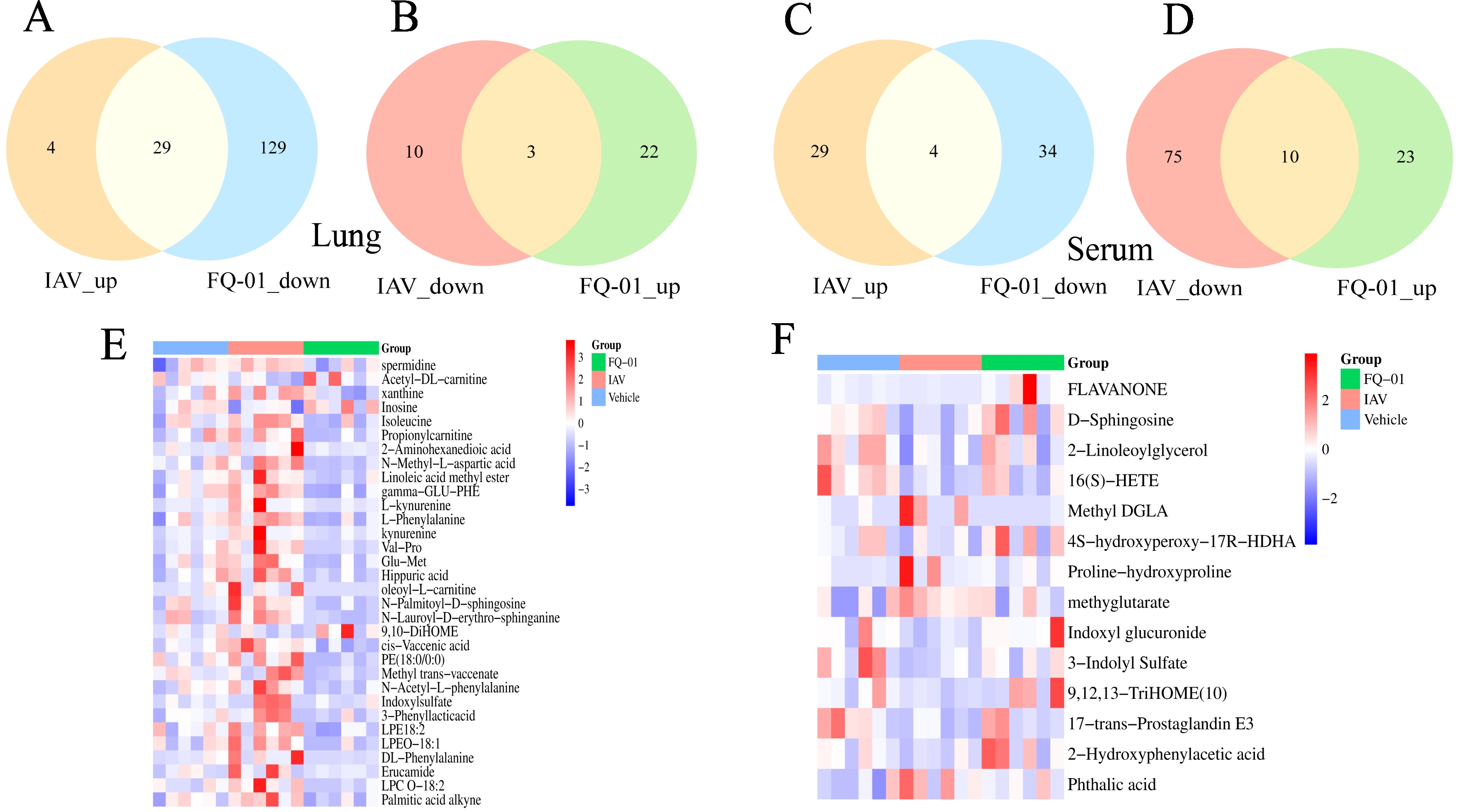

Supplement: Supplementary file 4 — Supplementary material 4. Analysis of serum and lung tissue differential metabolites in mice. (A) The DMs venn diagram of FQ-01 positive regulation in lung tissue. (B) The DMs venn diagram of FQ-01 negative regulation in lung tissue. (C) The DMs venn diagram of FQ-01 positive regulation in serum. (D) The DMs venn diagram of FQ-01 negative regulation in serum. (E) Heatmap of DMs in lung tissue. (F) Heatmap of DMs in serum. [file 13020_2025_1211_MOESM4_ESM.jpg]
