# Supplementary material for: Deciphering the antiviral mechanisms of Fangqin Qinggan decoction against influenza A virus: a multi-omics and machine learning approach
Source: Chin Med. 2025 Oct 5;20:158. doi: 10.1186/s13020-025-01211-0 (PMC12497347; doi:10.1186/s13020-025-01211-0)
Supplement: Supplementary file 9 — Supplementary material 9 [file 13020_2025_1211_MOESM9_ESM.docx]

**Supplementary data**

**1. Table 1 presents detailed information on the medicinal materials in FQ-01.**

Table 1: Information on FQ-01 Medicinal Ingredients

| **Herbal name** | **Amount(g)** | | **Batch number** | **Place of production** |
| --- | --- | --- | --- | --- |
| Scutellaria baicalensis Georgi | | 54 | 2203149 | Sichuan，China |
| Astragalus membranaceus(Fisch.) Bunge | | 54 | 21110206 | Sichuan，China |
| Saposhnikovia divaricata | | 30 | 220612-11 | Sichuan，China |
| Paeoniae Rubra Radix | | 30 | 220422-11 | Sichuan，China |

**2. Quality assessment of FQ-01 by HRMS**

We employed HRMS to analyze the components of FQ-01 extract, the total ion current and product ion chromatograms of FQ-01 standards were acquired in positive ion mode (Supplementary Figure 1), revealing detectable components include 5-O-methylvisammioside, Prim-O-glucosylcimifugin, baicalin, baicalein, and wogonin glucoside. Additionally, paeoniflorin was identified in negative ion mode. The concentrations of these compounds were as follows: 3.89 mg/g (5-O-methylvisammioside), 6.18 mg/g (Prim-O-glucosylcimifugin), 5.08 mg/g (baicalin), 81.55 mg/g (baicalein), 58.51 mg/g (wogonin glucoside), and 2.06 mg/g (paeoniflorin). Detailed values were presented in table 2.

Table 2: Quantification of identified active components in FQ-01

| **Compound name** | **Formula** | **Retention time (min)** | **Concentration (mg/g)** |
| --- | --- | --- | --- |
| 5-O-Methylvisammioside | C22H28O10 | 17.57 | 3.89 |
| Prim-O-glucosylcimifugin | C22H28O11 | 12.60 | 6.18 |
| Baicalein | C15H10O5 | 28.13 | 81.55 |
| Baicalin | C21H18O11 | 21.45 | 5.08 |
| Calycosin-7-glucoside | C22H22O10 | 14.17 | 58.51 |
| Paeoniflorin | C23H28O11 | 11.34 | 2.06 |

3. The primer sequences for the M gene of H1N1, as well as for the genes of pro-inflammatory factors (IL-1β, IL-6, and TNF-α) and key genes (myd88 and ccl5), are presented in table 3.

Table 3: Primer Sequences

| **Gene name** | **Primer Sequence** |
| --- | --- |
| α-Tubulin (F) | ATCACAGGCAAGGAGGATGC |
| α-Tubulin (R) | GCACTGGTCAGCCAGCTT |
| M (F) | CTTCTAACCGAGGTCGAAACGTA |
| M (R) | GGTGACAGGATTGGTCTTGTCTTTA |
| IL-6 (F) | TAGTCCTTCCTACCCCAATTTCC |
| IL-6 (R) | TGGTCCTTAGCCACTCCTTC |
| TNF-α (F) | GTGCCTATGTCTCAGCCTCTTCTC |
| TNF-α (R) | CCGATCACCCCGAAGTTCAGTAG |
| IL-1β (F) | CTGGTGTGTGACGTTCCCATTA |
| IL-1β (R) | CCGACAGCACGAGGCTTT |
| Myd88 (F) | AGCAGAACCAGGAGTCCGAGAAG |
| Myd88 (R) | GGTGATGCCTCCCAGTTCCTTTG |
| Ccl5 (F) | GACACCACTCCCTGCTGCTTTG |
| Ccl5 (R) | CTCTGGGTTGGCACACACTTGG |
